# Unfavorable genetic correlations between fecal egg count and milk production traits in the French blond-faced Manech dairy sheep breed

**DOI:** 10.1186/s12711-022-00701-1

**Published:** 2022-02-16

**Authors:** Sophie Aguerre, Jean-Michel Astruc, Andrés Legarra, Léa Bordes, Françoise Prevot, Christelle Grisez, Corinne Vial Novella, Francis Fidelle, Philippe Jacquiet, Carole Moreno-Romieux

**Affiliations:** 1 GenPhySE, UMR 1388, INRAE, ENVT, Université de Toulouse, 31326 Castanet-Tolosan, France; 2 Institut de l’Elevage, 31321 Castanet-Tolosan, France; 3 UMR INRAE-ENVT 1225, Interactions Hôtes-Agents Pathogènes, UMT Pilotage de la Santé des Ruminants, 31076 Toulouse, France; 4 CDEO, Quartier Ahetzia, 64130 Ordiarp, France

## Abstract

**Background:**

Genetic selection has proven to be a successful strategy for the sustainable control of gastrointestinal parasitism in sheep. However, little is known on the relationship between resistance to parasites and production traits in dairy breeds. In this study, we estimated the heritabilities and genetic correlations for resistance to parasites and milk production traits in the blond-faced Manech breed. The resistance to parasites of 951 rams from the selection scheme was measured through fecal egg counts (FEC) at 30 days post-infection under experimental conditions. Six milk production traits [milk yield (MY), fat yield (FY), protein yield (PY), fat content (FC), protein content (PC) and somatic cell score (LSCS)], were used in this study and were collected on 140,127 dairy ewes in first lactation, as part of the official milk recording. These ewes were related to the 951 rams (65% of the ewes were daughters of the rams).

**Results:**

Fecal egg counts at the end of the first and second infections were moderately heritable (0.19 and 0.37, respectively) and highly correlated (0.93). Heritabilities were moderate for milk yields (ranging from 0.24 to 0.29 for MY, FY and PY) and high for FC (0.35) and PC (0.48). MY was negatively correlated with FC and PC (− 0.39 and − 0.45, respectively). FEC at the end of the second infection were positively correlated with MY, FY and PY (0.28, 0.29 and 0.24, respectively with standard errors of ~ 0.10). These slightly unfavorable correlations indicate that the animals with a high production potential are genetically more susceptible to gastrointestinal parasite infections. A low negative correlation (− 0.17) was also found between FEC after the second infection and LSCS, which suggests that there is a small genetic antagonism between resistance to gastrointestinal parasites and resistance to mastitis, which is another important health trait in dairy sheep.

**Conclusions:**

Our results indicate an unfavorable but low genetic relationship between resistance to gastrointestinal parasites and milk production traits in the blond-faced Manech breed. These results will help the breeders’ association make decisions about how to include resistance to parasites in the selection objective.

**Supplementary Information:**

The online version contains supplementary material available at 10.1186/s12711-022-00701-1.

## Background

Gastrointestinal nematodes (GIN) are one of the major health issues in sheep breeding worldwide. They are responsible for important economic losses both directly due to the cost of mortalities and anthelmintic treatments, and indirectly due to their impact on production traits. On average, production losses of 15, 10 and 22% have been reported for weight gain, wool production and milk yield, respectively [1]. The extensive use of anthelmintic treatments as the only control strategy has been questioned for several years due to the development of resistance to the main molecules used in the treatments of parasitic infections [2].

In France, resistance to gastrointestinal parasites is an important issue for the sheep industry, particularly in the Pyrénées-Atlantiques, which is the second largest area of sheep milk production in the country. Its mild and humid climate creates favorable conditions for the development of GIN. Several cases of resistance to benzimidazoles have been identified in this area [3] and more recently, multi-resistance of *Haemonchus contortus* to ivermectin and benzimidazoles [4] and to eprinomectin and benzimidazoles [5] has been detected. In addition, only one anthelmintic drug (eprinomectin) is allowed during lactation with no withdrawal period for milk. Therefore, it is urgent to implement new strategies to complement the use of anthelmintics in the Pyrénées-Atlantiques area.

The feasibility and efficiency of genetic selection for resistance to GIN using fecal egg count (FEC) as an indicator trait for resistance to GIN have been demonstrated in the blond-faced Manech breed [6]. However, before including resistance to GIN as a new trait in the breeding objective, it is necessary to better understand the relationship between this trait and the production traits currently under selection.

Our objective was to estimate the genetic correlations between resistance to GIN (measured by FEC on rams that were experimentally-infected by *H. contortus*) and milk production traits that were measured on ewes including the daughters of the rams [milk yield (MY), fat and protein yields (FY and PY), fat and protein contents (FC and PC) and lactation mean somatic cell score (LSCS)].

## Methods

### Parasite resistance phenotypes

Between 2008 and 2018, most of the candidate rams that became the sires of ewes in the official milk recording system were phenotyped for resistance to GIN. The same protocol was carried out each year to phenotype the new candidate rams. In total, 951 blond-faced Manech rams from the breeding organization ‘Centre Départemental pour l’Elevage Ovin (CDEO)’ were phenotyped. Since the candidate rams were housed exclusively indoors on the farms where they were born and then at the CDEO, they were parasite-naïve before the first infection. Among the phenotyped rams, 90% were 2 or 3 years old and 10% were 4 to 9 years old at the time of the challenge.

The rams were challenged with two successive experimental infections with the “Humeau” strain of *H. contortus*. Each infection lasted 30 days and a 15-day recovery period separated the two infections. During the first years of phenotyping, different doses were used to determine those that resulted in sufficient variability between rams without too much impact on their health (maximum authorized hematocrit loss of 12%) or their semen production. The doses used in the first and second infections are described in Additional file 1: Table S1. An infection design including a first infection dose of 3500 larvae and a second infection dose of 5000 larvae was chosen to phenotype the candidate rams in the following years. Fecal samples were collected at the end of each infection. FEC was measured in the feces by the modified McMaster technique [7]. Then, the rams were drenched orally with Ivermectine (0.2 mg/kg of body weight, Oramec^©^ Merial) to stop the infection.

### Milk production phenotypes

Data for six economically important milk production traits were analyzed: milk yield per lactation (MY), fat (FY) and protein (PY) yields per lactation, lactation mean fat (FC) and protein (PC) contents and lactation mean SCS (LSCS).

The data were obtained from the official milk records. The following milk recording protocol is applied in the blond-faced Manech breed: milk yield is measured individually 4 to 6 times per lactation and the composition of the milk is analyzed from samples taken at 2 to 4 test-days among the first four test-days during the first lactation. ICAR guidelines were followed [8].

Lactation performances were estimated from these test-day records as described by Rupp et al. [9]. MY was estimated using the Fleischmann method and adjusted for milking length on a reference period of 160 days [10]. FC and PC were calculated as the arithmetic mean of test-day records adjusted for days in milk (DIM). FY and PY were computed as the product of MY by FC and PC, respectively. Test-day somatic cell counts (SCC) were log-transformed to somatic cell scores (SCS). LSCS were estimated as the weighted arithmetic mean of test-day SCS adjusted for DIM.

Milk recording data for 140,127 dairy ewes in first lactation during the 2009–2018 period were included in the analyses. These 10 years included on-farm performances of females in first lactation that were born from rams phenotyped for resistance to GIN.

### Pedigree information

All candidate rams that were phenotyped for resistance to GIN were closely related. They were born through artificial insemination (AI) from dams of sires and sires of sires (25 to 30 individuals, each year) that were selected from the selection nucleus. These rams were also closely related to the ewes that were phenotyped for milk production traits through AI. About 65% of the ewes that were phenotyped for milk production traits were the progeny of the rams that were phenotyped for resistance to GIN. Among the 951 rams phenotyped for resistance to GIN, 729 (77%) had more than 20 daughters with on-farm performances for MY, FY, PY, FC, PC and SCC (see Additional file 2: Table S2). The other rams did not have offspring with on-farm performances because they were too young at the time of phenotyping to already have progeny in the official milk control system but they were related to the other rams through AI as mentioned above.

Pedigree information across five generations was used for the estimation of the genetic parameters presented below and included 217,467 individuals.

### Statistical analyses

Two computational methods were used to estimate the genetic correlations between resistance to GIN and milk production traits. A first method included data resistance to GIN collected on the males and data on milk traits collected on the females, whereas the second method included daughter yield deviations (DYD) of the males for milk traits. The genetic and residual variances were computed in bivariate analyses by average information restricted maximum likelihood. The analyses were performed with the AIREMLF90 program [11].

#### Method 1

Heritabilities and genetic correlations were estimated for each measure of FEC (one after each of two successive experimental infections) and the six milk traits using bivariate analyses, which exploit the close pedigree relationship between the rams measured for resistance to GIN and the ewes measured for milk traits.

FEC were fourth-root transformed in order to normalize the data before subsequent analyses (root_FEC_inf1 for FEC at the end of the first infection and root_FEC_inf2 for FEC at the end of the second infection). Fourth-root transformation was selected because it results in the best normalization of the data.

The model for FEC traits was:

$${\mathrm{y}}_{\mathrm{ij}}=\upmu +{\mathrm{cg}}_{\mathrm{i}}+{\mathrm{age}}_{\mathrm{j}}+{\mathrm{a}}_{\mathrm{j}}+{\mathrm{e}}_{\mathrm{ij}},$$

where ${\mathrm{y}}_{\mathrm{ij}}$ is the trait (root_FEC_inf1 or root_FEC_inf2), $\upmu$ is the population mean, ${\mathrm{cg}}_{\mathrm{i}}$ is the contemporary group $\mathrm{i}$ (combination of the year of measure and dose effects, 9 levels: year (2008) × doses (5000–5000); year (2008) × doses (7500–7500); year (2009) × doses (3500–5000); year (2011) × doses (3000–3000); year (2013) × doses (3500–5000); year (2015) × doses (3500–5000); year (2016) × doses (3500–5000); year (2017) × doses (3500–5000); year (2018) × doses (3500–5000)), ${\mathrm{age}}_{\mathrm{j}}$ is the fixed effect of the age of individual $\mathrm{j}$ at the time of sampling (4 levels: 1 year; 2 years; 3 years; 4 years and older), ${\mathrm{a}}_{\mathrm{j}}$ is the additive genetic random effect of individual $\mathrm{j}$ following a normal distribution with mean 0 and variance ${\sigma }_{u}^{2}$ and ${\mathrm{e}}_{\mathrm{ij}}$ is the random residual effect.

The milk traits data were fitted according to the following models:

$${\mathrm{MY}}_{\mathrm{ijklm}}=\upmu +{\mathrm{hy}}_{\mathrm{i}}+{\mathrm{age}}_{\mathrm{j}}+{\mathrm{ml}}_{\mathrm{k}}+{\mathrm{int}}_{\mathrm{l}}+{\mathrm{a}}_{\mathrm{n}}+{\mathrm{e}}_{\mathrm{ijkln}},$$

$${\mathrm{FY}}_{\mathrm{ijmn}}=\upmu +{\mathrm{hy}}_{\mathrm{i}}+{\mathrm{age}}_{\mathrm{j}}+{\mathrm{c}}_{\mathrm{m}}+{\mathrm{a}}_{\mathrm{n}}+{\mathrm{e}}_{\mathrm{ijmn}},$$

$${\mathrm{PY}}_{\mathrm{ijmn}}=\upmu +{\mathrm{hy}}_{\mathrm{i}}+{\mathrm{age}}_{\mathrm{j}}+{\mathrm{c}}_{\mathrm{m}}+{\mathrm{a}}_{\mathrm{n}}+{\mathrm{e}}_{\mathrm{ijmn}},$$

$${\mathrm{FC}}_{\mathrm{ijmn}}=\upmu +{\mathrm{hy}}_{\mathrm{i}}+{\mathrm{age}}_{\mathrm{j}}+{\mathrm{c}}_{\mathrm{m}}+{\mathrm{a}}_{\mathrm{n}}+{\mathrm{e}}_{\mathrm{ijmn}},$$

$${\mathrm{PC}}_{\mathrm{ijmn}}=\upmu +{\mathrm{hy}}_{\mathrm{i}}+{\mathrm{age}}_{\mathrm{j}}+{\mathrm{c}}_{\mathrm{m}}+{\mathrm{a}}_{\mathrm{n}}+{\mathrm{e}}_{\mathrm{ijmn}},$$

$${\mathrm{LSCS}}_{\mathrm{ijkn}}=\upmu +{\mathrm{hy}}_{\mathrm{i}}+{\mathrm{age}}_{\mathrm{j}}+{\mathrm{ml}}_{\mathrm{k}}+{\mathrm{a}}_{\mathrm{n}}+{\mathrm{e}}_{\mathrm{ijkn}},$$

where ${\mathrm{hy}}_{\mathrm{i}}$ is the fixed effect of flock × year $\mathrm{i}$ (2229 levels), ${\mathrm{age}}_{\mathrm{j}}$ is the fixed effect of age at first lambing $\mathrm{j}$ (4 levels: age < 300 days or age > 1279 days or unknown age; 299 days < age < 600 days; 599 days < age < 930 days; 929 days < age < 1280 days), ${\mathrm{ml}}_{\mathrm{k}}$ is the fixed effect of month at lambing $\mathrm{k}$ (6 levels: January; February; March; April–May–June–July; August–September–October–November; December), ${\mathrm{int}}_{\mathrm{l}}$ is the fixed effect of interval between lambing and first-test day $\mathrm{l}$ (5 levels: int < 25 days; 24 days < int < 40 days; 39 days < int < 50 days; 49 days < int < 65 days; 64 days < int < 86 days), ${\mathrm{c}}_{\mathrm{m}}$ is the fixed effect (used to model milk composition) of combination $\mathrm{m}$ of the test-day (TD) when sampling is done (6 levels: e.g. ${\mathrm{TD}}_{1}+{\mathrm{TD}}_{2}+{\mathrm{TD}}_{3}$; ${\mathrm{TD}}_{2}+{\mathrm{TD}}_{3}+{\mathrm{TD}}_{4}$; ${\mathrm{TD}}_{2}+{\mathrm{TD}}_{3}$; ${\mathrm{TD}}_{1}+{\mathrm{TD}}_{2}$ …), ${\mathrm{a}}_{\mathrm{n}}$ is the additive genetic random effect of individual $\mathrm{n}$ and ${\mathrm{e}}_{\mathrm{ijkln}}$, ${\mathrm{e}}_{\mathrm{ijmn}}$ and ${\mathrm{e}}_{\mathrm{ijkn}}$ are the random residual effects.

#### Method 2

In this method, additive genetic effects were fitted for the candidate rams only. For resistance to GIN, the same dataset and model as for Method 1 were used. For milk production traits, DYD were computed using data from the national genetic evaluation and the models presented in Method 1. DYD correspond to the average performance of the female offspring of a ram corrected for environmental effects and the genetic value of the dams [12].

## Results

The basic statistics for the traits measured on the blond-faced Manech rams are in Table 1. The 951 experimentally-infected rams excreted a 20% lower number of eggs upon reinfection than in the first infection when they were naïve towards *H. contortus* (2567 and 2064 eggs per g at the end of the first and second infection, respectively). This was also observed in other studies using experimental protocols of infection that were similar to ours [13, 14]. For the ewes in first lactation, the average milk yield corrected for lactation length and expressed in mature equivalent was 261 L/year. Average fat and protein contents were 61.61 g/L and 48.88 g/L, respectively, which are low because sampling was performed in the morning at mid-lactation.

**Table 1:** Descriptive statistics on the rams phenotyped for parasite resistance and the ewes phenotyped for milk production traits

Variable	Number of animals	Mean	SD	Minimum	Maximum
Parasite resistance traits—Males in control station					
FEC_inf1_T0 (eggs/g)	951	0	0	0	0
FEC_inf1 (eggs/g)	944	2567	1886	0	11,400
FEC_inf2 (eggs/g)	912	2064	1983	0	14,900
root_FEC_inf1	944	6.5	1.9	0	10.3
root_FEC_inf2	912	5.9	2.1	0	11.0
Milk production traits—Milk recording data					
MY (L/year)	139,496	261	76	29	495
FY (kg/year)	139,378	15.98	4.81	1.32	30.95
PY (kg/year)	139,507	12.74	3.80	1.20	24.48
FC (g/L)	139,226	61.61	8.29	35.78	87.71
PC (g/L)	139,376	48.88	4.23	35.90	62.06
LSCS	138,863	8.8	0.2	8.1	9.4

SD: standard deviation; FEC_inf1_T0: fecal egg count at the day of the first infection; FEC_inf1: fecal egg count at the end of the first infection; FEC_inf2: fecal egg count at the end of the second infection; root_FEC_inf1 and root_FEC_inf2 are the fourth-root transformed values for FEC_inf1 and FEC_inf2 respectively; MY: milk yield; FY: fat yield; PY: protein yield; FC: fat content; PC: protein content; LSCS: lactation mean somatic cell score

The estimates of heritability and genetic correlations are in Table 2. The results obtained with the DYD were very similar to those obtained with the full data (see Additional file 3: Table S3 and Additional file 4: Table S4). The heritabilities of FEC were moderate at the end of the first infection (0.19) and higher at the end of the second infection (0.37). We also found a high genetic correlation of 0.93 between root_FEC_inf1 and root_FEC_inf2. The heritabilities of MY, FY and PY (0.29, 0.24 and 0.26, respectively) and FC and PC (0.35 and 0.48, respectively) were moderate to high; whereas the heritability of LSCS was lower (0.15). The estimates of the genetic variances are in Additional file 5: Table S5. Yield measures (MY, FY, and PY) were highly and positively correlated with genetic correlations ranging from 0.83 to 0.91. In contrast, the genetic correlations between MY and FC and PC were negative, showing a genetic antagonism between these traits. Finally, a slightly unfavorable genetic correlation was observed between MY and LSCS (0.14). The genetic correlations of root_FEC_inf1 with the milk traits were not significant. However, we found unfavorable genetic correlations of root_FEC_inf2 with MY (0.28), FY (0.29), PY (0.24) and LSCS (− 0.17), and a favorable negative genetic correlation of root_FEC_inf2 with PC (− 0.2).

**Table 2:** Genetic parameters for parasite resistance and milk production traits

Traits	root_FEC_inf1	root_FEC_inf2	MY	FY	PY	FC	PC	LSCS
root_FEC_inf1	*0.19* ± *0.07*	0.93 ± 0.25	− 0.14 ± 0.24	0.19 ± 0.19	− 0.20 ± 0.27	0.57 ± 0.73	0.03 ± 0.18	− 0.05 ± 0.20
root_FEC_inf2		*0.37* ± *0.09*	0.28 ± 0.10	0.29 ± 0.11	0.24 ± 0.10	− 0.02 ± 0.09	− 0.20 ± 0.10	− 0.17 ± 0.11
MY			*0.29* ± *0.007*	0.83 ± 0.006	0.91 ± 0.003	− 0.39 ± 0.02	− 0.45 ± 0.01	0.14 ± 0.02
FY				*0.24* ± *0.007*	0.85 ± 0.005	0.18 ± 0.02	− 0.15 ± 0.02	0.21 ± 0.03
PY					*0.26* ± *0.007*	− 0.18 ± 0.02	− 0.04 ± 0.02	0.21 ± 0.02
FC						*0.35* ± *0.007*	0.54 ± 0.01	0.09 ± 0.02
PC							*0.48* ± *0.007*	0.08 ± 0.02
LSCS								*0.15* ± *0.006*

Heritability estimates are written in italics on the diagonal and genetic correlation estimates are above the diagonal. These estimates are considered significantly different to zero when zero is out of their confidence interval (twice the standard error of the estimates), which corresponds roughly to a p-value lower than 0.05

Root_FEC_inf1 and root_FEC_inf2 are the fourth-root transformed values for FEC_inf1 and FEC_inf2 respectively; MY: milk yield; FY: fat yield; PY: protein yield; FC: fat content; PC: protein content; LSCS: lactation mean somatic cell score

## Discussion

In this paper, we report the estimates of genetic parameters for six milk production traits (MY, FY, PY, FC, PC, and LSCS) and for resistance to GIN (root_FEC_inf1 and root_FEC_inf2). The heritability values estimated for root_FEC_inf1 and root_FEC_inf2 were consistent with the estimates (0.2 to 0.4) reported in the literature for other breeds [15–18]. The high genetic correlation between these two measures of FEC under experimental conditions of infestation was also consistent with other studies [18, 19]. The genetic parameters estimated for milk production traits are within the range of values that are classically observed in dairy breeds (see review by Carta et al. [20]). The values were consistent with the genetic parameters for milk production traits found in the Lacaune breed [21] and in the Sarda breed [22] but not with estimates reported for Spanish breeds. Indeed, lower heritabilities for MY, FY, PY, FC and PC have been estimated in Latxa black-faced sheep [23] and negative genetic correlations between MY and LSCS have been reported in the Spanish Churra [24] and in the Latxa black-faced breeds [25]. The differences observed between the French and Spanish breeds might be explained by the different methods used for recording milk production traits as discussed by Legarra and Ugarte [23], i.e. two different methods (AT and AC) as described in the ICAR guidelines [8] are used in Spain and France, respectively. The AT method with alternate morning/evening milk recordings is used for the Spanish breeds whereas the AC method that measures milk at the same milking at each recording visit is used for the French breeds. These differences in milk recording that impact milk yield and milk composition measures might have an effect on the genetic parameters estimates.

Most studies that aim at estimating genetic parameters of resistance to GIN are carried out in natural conditions of infection which may lead to variable results. Using a modeling approach, Doeschl-Wilson et al. [26] showed that the genetic correlation between body weight and FEC can vary over time, which underlies the importance of the time at which sampling is done after infection. Infection pressure also seems to have an impact on the estimated genetic parameters. Doeschl-Wilson et al. [26] also found that, under natural conditions of infection, the correlation of meat production traits with FEC was low in environments where FEC were low (assuming a low infection pressure), whereas it increased in environments where FEC were higher (assuming a high infection pressure). Such different results obtained under natural conditions of infection lead to a lack of consensus between genetic parameter estimation studies. In 2005, Safari et al. [27] reported genetic correlations ranging from − 0.17 to 0.21 between FEC and wool production traits and from − 0.63 to 0.24 between FEC and meat production traits in sheep. Although few studies have been conducted on small dairy ruminants, two on Saanen goats should be mentioned: Heckendorn et al. [28] estimated a genetic correlation of 0.49 between FEC and milk yield, whereas Morris et al. [29] found non-significant genetic correlations between FEC and milk traits. However, since these two studies were conducted under natural conditions of infection, the limitations discussed above may apply and could explain the divergence between the results.

The originality of our study lies in the use of experimental infections to investigate the relationship between milk production and resistance to GIN. Our experimental protocol of infection allows us to control precisely the infection pressure and the time of sampling, thus avoiding the previously mentioned biases. We found slightly unfavorable genetic correlations between root_FEC_inf2 and milk traits (MY, PY, FY and FC), which might indicate competition between milk production and immune response.

Moreover, our experimental protocol included two experimental infections that were performed on naïve animals at the time of the first infection. The first infection triggers an innate response and the initiation of the adaptive response. This response is characterized by the key role of physical barriers such as mucus production and muscle contractility that prevent the infective larvae from passing into the abomasum [30]. The second infection mimics reinfections in the animals during their successive grazing seasons. After this second infection, we observed that the rams developed an effective adaptive immune response to the infection. Thus, the protein cost associated with resistance to GIN can be higher during the second infection and could affect resource allocation for production traits such as MY, PY, FY and FC. This hypothesis could explain the unfavourable genetic correlations that we estimated in this work.

The impact of genetic selection for a better resistance to GIN on the ability of the animals to cope with other diseases is another matter of interest. Along with GIN infections, mastitis is a major health constraint in dairy sheep production and is responsible for an altered quality of milk and a higher flock renewal. Studies performed on ewes in Greece showed that GIN infections might be a predisposing factor to clinical mastitis, since ewes infected by parasites in experimental conditions were more likely to develop clinical mastitis than non-infected ewes [31]. These findings were confirmed in field conditions where a higher prevalence of subclinical mastitis was observed among infected ewes, with the prevalence being higher in ewes with high FEC than in ewes with low FEC [32]. Under natural conditions of parasite and mastitis infections, Sechi et al. [33] estimated a zero phenotypic correlation and a favorable genetic correlation (0.21) in a Sardinian × Lacaune backcross. In our study, the genetic correlation between FEC and LSCS was not significantly different from 0, which agrees with the results reported by Rupp et al. [34] who observed no significant difference in FEC measured after a challenge with *H. contortus* in goats from divergent lines selected on SCS. These results suggest that genetic selection for low FEC should either slightly impact or not impact resistance to mastitis.

The effect of the unfavorable genetic correlation between FEC and milk production traits might lead to a deterioration of the resistance to GIN for which we provide here an order of magnitude. Roughly, the genetic progress is ΔG = 0.25 standard deviations of the breeding objective (composed of MY and milk contents as well as LSCS since 2017) per year. Assuming that the only selected trait is MY, a genetic progress of 25% on this trait would lead to a deterioration of 0.003% for FEC traits, which is quite low but can lead to a real deterioration of the resistance to GIN after many years of selection. It is important to take these correlations into account in order to avoid increasing even more the deterioration of the resistance to GIN. However, since the correlations are not too high, it is possible to select both on milk production traits and resistance to GIN.

## Conclusions

We found unfavorable but low genetic correlations between milk production traits and resistance to parasite in blond-faced Manech dairy sheep, which will help the breeders’ association (CDEO) of this breed make decisions about how to include FEC traits in their global breeding objective.

## Supplementary Information

**Additional file 1: Table S1.** Doses of infective larvae given to the rams on the control station. This table contains information about the larvae dose by year and the number of rams by year and dose

**Additional file 2: Table S2.** Number of offspring per ram. This table contains the description of the distribution of offspring among the 951 rams.

**Additional file 3: Table S3.** Descriptive statistics for the daughter yield deviations of the 951 rams: mean, standard deviation, minimum and maximum for the daughter yield deviation of 951 rams

**Additional file 4: Table S4.** Genetic correlations between fecal egg counts and milk production traits estimated by using the fecal egg counts and the DYD of the rams. Genetic correlations were calculated on the ram population (951 animals) using calculated DYD and measured FEC in the AIREML software.

**Additional file 5: Table S5.** Genetic and residual variances for parasite resistance and milk production calculated using the AIREML software.

## Data Availability

The datasets used and analyzed during this study are available from the corresponding author upon reasonable request.
