# Supplementary material for: Unfavorable genetic correlations between fecal egg count and milk production traits in the French blond-faced Manech dairy sheep breed
Source: Genet Sel Evol. 2022 Feb 16;54:14. doi: 10.1186/s12711-022-00701-1 (PMC8848663; doi:10.1186/s12711-022-00701-1)
Supplement: Supplementary file 1 — Additional file 1: Table S1. Doses of infective larvae given to the rams on the control station. This table contains information about the larvae dose by year and the number of rams by year and dose [file 12711_2022_701_MOESM1_ESM.docx]

Additional file 1: Table S1. Doses of infective larvae given to the rams in control station

| Year | Doses | | Number of rams |
| --- | --- | --- | --- |
|  | Infection 1 | Infection 2 |  |
| 2008 | 5000 | 5000 | 30 |
|  | 7500 | 7500 | 19 |
| 2009 | 3500 | 5000 | 99 |
| 2011 | 3000 | 3000 | 52 |
| 2013 | 3500 | 5000 | 132 |
| 2015 | 3500 | 5000 | 130 |
| 2016 | 3500 | 5000 | 132 |
| 2017 | 3500 | 5000 | 124 |
| 2018 | 3500 | 5000 | 239 |
