# Supplementary material for: Unfavorable genetic correlations between fecal egg count and milk production traits in the French blond-faced Manech dairy sheep breed
Source: Genet Sel Evol. 2022 Feb 16;54:14. doi: 10.1186/s12711-022-00701-1 (PMC8848663; doi:10.1186/s12711-022-00701-1)
Supplement: Supplementary file 2 — Additional file 2: Table S2. Number of offspring per ram. This table contains the description of the distribution of offspring among the 951 rams. [file 12711_2022_701_MOESM2_ESM.docx]

Additional file 2: Table S2. Number of offspring per ram

| Number of daughters per ram | Number of rams |
| --- | --- |
| 0 - 20 | 21 |
| 20 - 50 | 108 |
| 50 - 100 | 324 |
| 100 - 200 | 144 |
| 200 - 500 | 82 |
| > 500 | 28 |
