# Supplementary material for: Unfavorable genetic correlations between fecal egg count and milk production traits in the French blond-faced Manech dairy sheep breed
Source: Genet Sel Evol. 2022 Feb 16;54:14. doi: 10.1186/s12711-022-00701-1 (PMC8848663; doi:10.1186/s12711-022-00701-1)
Supplement: Supplementary file 3 — Additional file 3: Table S3. Descriptive statistics for the daughter yield deviations of the 951 rams: mean, standard deviation, minimum and maximum for the daughter yield deviation of 951 rams [file 12711_2022_701_MOESM3_ESM.docx]

Additional file 3: Table S3. Descriptive statistics for the daughter yield deviations of the rams

|  |  |  |  |  |  |
| --- | --- | --- | --- | --- | --- |
| Variable | Number of rams | Mean | Standard deviation | Minimum | Maximum |
| DYD_MY | 639 | 286.76 | 108.95 | -46.44 | 672.50 |
| DYD_FY | 639 | 7.88 | 7.90 | -14.39 | 33.74 |
| DYD_PY | 639 | 6.12 | 5.77 | -12.69 | 26.02 |
| DYD_FC | 639 | 0.37 | 2.34 | -5.99 | 7.50 |
| DYD_PC | 639 | 0.17 | 1.15 | -3.19 | 4.39 |
| DYD_LSCS | 639 | 56.34 | 283.22 | -718.24 | 1129.71 |
|  |  |  |  |  |  |
|  |  |  |  |  |  |

DYD_MY: daughter yield deviation for milk yield
DYD_FY: daughter yield deviation for fat yield
DYD_PY: daughter yield deviation for protein yield
DYD_FC: daughter yield deviation for fat content
DYD_PC: daughter yield deviation for protein content
DYD_LSCS: daughter yield deviation for log transformed somatic cell score
