# Supplementary material for: Unfavorable genetic correlations between fecal egg count and milk production traits in the French blond-faced Manech dairy sheep breed
Source: Genet Sel Evol. 2022 Feb 16;54:14. doi: 10.1186/s12711-022-00701-1 (PMC8848663; doi:10.1186/s12711-022-00701-1)
Supplement: Supplementary file 4 — Additional file 4: Table S4. Genetic correlations between fecal egg counts and milk production traits estimated by using the fecal egg counts and the DYD of the rams. Genetic correlations were calculated on the ram population (951 animals) using calculated DYD and measured FEC in the AIREML software. [file 12711_2022_701_MOESM4_ESM.docx]

Additional file 4: Table S4. Genetic correlations between fecal egg counts and milk production traits estimated using the fecal egg counts and the DYD of the rams.

|  |  |  | |  |  |  |  |  |  |
| --- | --- | --- | --- | --- | --- | --- | --- | --- | --- |
|  | root_FEC_inf1 | root_FEC_inf2 | DYD_MY | | DYD_FY | DYD_PY | DYD_FC | DYD_PC | DYD_LSCS |
| root_FEC_inf1 |  | 0.93 ± 0.25 | | -0.18 ± 0.09 | NS | -0.28 ± 0.11 | NS | NS | NS |
| root_FEC_inf2 |  |  | | 0.27 ± 0.04 | 0.22 ± 0.07 | 0.17 ± 0.09 | NS | -0.19 ± 0.10 | NS |
| DYD_MY |  |  | |  | 0.71 ± 0.02 | 0.79 ± 0.02 | -0.28 ± 0.05 | -0.38 ± 0.04 | NS |
| DYD_FY |  |  | |  |  | 0.87 ± 0.02 | 0.23 ± 0.04 | -0.16 ± 0.04 | 0.15 ± 0.05 |
| DYD_PY |  |  | |  |  |  | -0.14 ± 0.05 | -0.08 ± 0.04 | 0.15 ± 0.05 |
| DYD_FC |  |  | |  |  |  |  | 0.46 ± 0.07 | 0.20 ± 0.10 |
| DYD_PC |  |  | |  |  |  |  |  | 0.21 ± 0.10 |
| DYD_LSCS |  |  | |  |  |  |  |  |  |
|  |  |  | |  |  |  |  |  |  |

1. root_FEC_inf1 and root_FEC_inf2 are the fourth-root transformed values for FEC_inf1 and FEC_inf2 respectively
   DYD_MY: daughter yield deviation for milk yield
   DYD_FY: daughter yield deviation for fat yield
   DYD_PY: daughter yield deviation for protein yield
   DYD_FC: daughter yield deviation for fat content
   DYD_PC: daughter yield deviation for protein content
   DYD_LSCS: daughter yield deviation for log transformed somatic cell score
2. The genetic correlation estimates are considered significantly different to zero when zero is out of their confidence interval (2 times the standard error of the estimates), which corresponds roughly to a p-value <0.05.
