# Supplementary material for: Unfavorable genetic correlations between fecal egg count and milk production traits in the French blond-faced Manech dairy sheep breed
Source: Genet Sel Evol. 2022 Feb 16;54:14. doi: 10.1186/s12711-022-00701-1 (PMC8848663; doi:10.1186/s12711-022-00701-1)
Supplement: Supplementary file 5 — Additional file 5: Table S5. Genetic and residual variances for parasite resistance and milk production calculated using the AIREML software. [file 12711_2022_701_MOESM5_ESM.docx]

Additional file 5: Table S5: Genetic and residual variances for parasite resistance and milk production

|  | root_FEC_inf1 | root_FEC_inf2 | MY (L²) | FY (kg²) | PY (kg²) | FC (g²/L²) | PC (g²/L²) | LSCS |
| --- | --- | --- | --- | --- | --- | --- | --- | --- |
| $\sigma_{a}^{2}$ | 0.50 | 1.69 | 964.24 | 3.20 | 1.84 | 18.66 | 6.15 | 0.0055 |
| $\sigma_{e}^{2}$ | 2.15 | 2.87 | 2312.2 | 10.18 | 5.31 | 34.74 | 6.63 | 0.03 |

root_FEC_inf1 and root_FEC_inf2 are the fourth-root transformed values for FEC_inf1 and FEC_inf2 respectively
MY: milk yield
FY: fat yield
PY: protein yield
FC: fat content
PC: protein content
LSCS: lactation mean somatic cell score
